# Management of a Massive Retropharyngeal Abscess Through an External Cervical Approach: A Case Report

**DOI:** 10.7759/cureus.98740

**Published:** 2025-12-08

**Authors:** Shikhah Alzayed, Ahmed AlOlaywi

**Affiliations:** 1 Department of Otorhinolaryngology - Head and Neck Surgery, Aljouf Health Cluster, Aljouf, SAU

**Keywords:** adolescent, corticosteroid therapy, deep neck space infections, external cervical approach, retropharyngeal abscess

## Abstract

Retropharyngeal abscess appears to be decreasing in incidence in older children. It can be caused by local trauma to the posterior pharynx. Contributing risk factors include poor oral hygiene, diabetes, immunocompromised states, and low socioeconomic status. Early diagnosis is essential, as it may lead to potentially life-threatening dyspnea that requires rapid airway management.

A 14-year-old boy was referred to our hospital with complaints of dyspnea, rapidly progressive dysphagia, and high-grade fever for four days. The patient reported a history of neck pain and stiffness over the past twenty days. Physical examination was remarkable for stridor, inspiratory wheezing, and mild lower neck swelling. A lateral neck radiograph was initially performed, followed by a neck CT with contrast conducted in the emergency department, which demonstrated a rim-enhancing retropharyngeal abscess measuring approximately 10.5 x 3.4 x 5.4 cm, extending from the C3 to T3 level, displacing the esophagus and trachea anteriorly with associated mild to moderate narrowing of the airway. A smaller abscess was also identified adjacent to the primary lesion, measuring approximately 5.3 x 1.3 x 0.8 cm, at the level of C5 to T2. The patient was admitted to the ICU under the care of the otorhinolaryngology department. The airway was maintained, and intravenous antibiotics and corticosteroids were initiated. Surgical incision and drainage of the abscess through a cervical approach were carried out. The wound was irrigated, and a 10-Fr closed-suction drain (BIOVAC™, Biometrix™ Critical Care Solutions, QMD - Quality Medical Devices, Slovakia) was inserted through the left anterior cervical incision, advanced 3 cm into the abscess cavity, and connected to continuous suction; the procedure was performed in conjunction with the thoracic surgery team. The patient was transferred to the ward, where he showed marked improvement in respiratory distress and was able to tolerate liquids, followed by a soft diet.

The administration of intravenous antibiotics and corticosteroid therapy, along with surgical incision and drainage of the retropharyngeal abscess, was effective in treating this patient’s condition and ended with marked clinical improvement.

## Introduction

Deep neck infections occur in potential spaces and fascial planes of the neck; the retropharyngeal abscess is one of the most common deep neck space infections [1]. Retropharyngeal abscesses are rare in adults; however, they are more frequent in younger children due to the abundance of retropharyngeal lymph nodes and immaturity of their immune system [2,3]. These abscesses can be triggered by oropharyngeal trauma or dental disease, although in younger children, upper respiratory tract infections commonly precede abscess formation. Patients presenting with retropharyngeal abscesses are typically febrile with symptoms of upper aerodigestive tract obstruction, including dysphagia, odynophagia, inability to tolerate oral secretions, neck stiffness, and respiratory distress [4]. Such abscesses need prompt diagnosis and early management, as they can lead to rapid airway compromise [2]. We report this case to highlight our medical and surgical treatment of a large retropharyngeal abscess that partially narrowed the airway.

## Case presentation

A 14-year-old boy with no known chronic illness was referred to our hospital, where he was admitted to the ICU for the management of acute respiratory distress. The patient reported a history of neck pain for the past twenty days and presented to the emergency department with complaints of dyspnea and rapidly progressive dysphagia, initially for solids and then later for both solids and liquids, had difficulty moving his neck, and also had a high-grade fever over the last four days. At presentation, his temperature was 39.4°C, respiratory rate 26/min, and oxygen saturation 94%. On physical examination, the patient was conscious and oriented, febrile, and had stridor with inspiratory wheezing on auscultation. No oropharyngeal erythema, tonsillar hypertrophy, or exudate was noted. Additionally, he had tenderness and mild swelling over the lower neck region.

**Figure 1:**
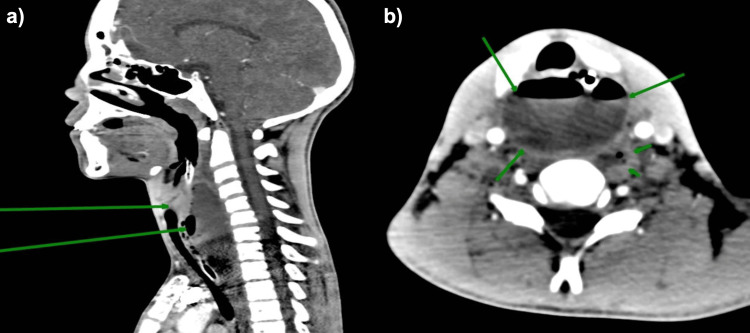
Neck CT angiography findings (a) Sagittal CT angiography view of the neck showing an extensive collection with pockets of gas and air-fluid level with enhancing walls in the retropharyngeal space extending from C3 to T3, displacing the trachea and compressing the esophagus. (b) Axial CT angiography view of the neck showing a larger abscess (long arrows) and a smaller abscess (short arrows). CT: computed tomography

The rapid response team was engaged in the emergency department upon the patient's arrival. After stabilization of the patient’s condition, the presence of dysphagia, neck stiffness, and fever, with marked dyspnea and audible stridor, raised our suspicion for a deep neck space infection. A lateral neck radiography was first performed, showing a widened prevertebral space with an air-fluid level. Then, a neck CT with contrast scan was performed, showing an extensive fluid collection with gas bubbles and an air-fluid level with enhancing walls in the retropharyngeal space, measuring approximately 10.5 x 3.4 x 5.4 cm, extending from the C3 to T3 level and displacing the esophagus as well as the trachea anteriorly with associated mild to moderate narrowing of the airway (Figure 1a-1b). Additionally, a smaller collection was identified adjacent to the primary lesion, measuring approximately 5.3 x 1.3 x 0.8 cm, at the C5 to T2 level (Figure 1b).

The patient was admitted to the ICU under the care of the otorhinolaryngology department with a diagnosis of retropharyngeal abscess. The airway track was maintained, and nebulization with dexamethasone was given only on the first day of admission. Intravenous antibiotics and corticosteroid therapy were initiated, and, based on the patient’s clinical course, adjustments were made later (Table 1). In the operating room, in collaboration with the thoracic surgery team, a hockey stick incision was made on the left side of the neck, anterior to the sternocleidomastoid muscle, and a large amount of foul-smelling pus was drained. Then the wound was irrigated, and a 10-Fr closed-suction drain (BIOVAC™, Biometrix™ Critical Care Solutions, QMD - Quality Medical Devices, Slovakia) was inserted percutaneously through the left anterior cervical incision, advanced 3 cm into the abscess cavity, connected to a bulb suction system, and secured externally with a 5-0 nylon suture. The patient was transferred to the ward, and on postoperative day four, the closed-suction drain was removed. The microbiological analysis revealed that the pharyngeal swab was positive for *Staphylococcus aureus* and *Staphylococcus epidermidis*. At the same time, no growth occurred in the aerobic culture of the exudate, and due to inadequate anaerobic transport and incubation conditions, the anaerobic culture could not be performed. The patient showed improvement and was discharged after seven days of hospitalization.

**Table 1:** Intravenous medication timeline

Day (from admission)	Phase	Medication	Dose	Route	Reason for modification
0	Initial therapy	Ampicillin sulbactam	1000 mg q8h	IV	Continued
0	Initial therapy	Vancomycin	1000 mg q8h	IV	Started, then replaced with imipenem–cilastatin due to an elevated vancomycin trough level
0	Initial therapy	Dexamethasone	8 mg q12h	IV	Completed (72h)
1	Postoperative therapy	Metronidazole	500 mg q8h	IV	Started postoperation immediately and continued
2	Adjusted therapy	Imipenem-cilastatin	500 mg q12h	IV	Continued

## Discussion

Retropharyngeal abscesses are rare in adults and more common in younger children, and their incidence tends to decrease in older children [2,3]. It is often polymicrobial, commonly involving Group A *Streptococcus* (*Streptococcus pyogenes*), *Staphylococcus aureus*, *Fusobacterium*, *Haemophilus*, and other respiratory anaerobic organisms. In adults and older children, retropharyngeal abscesses can result from trauma to the posterior pharynx, leading to infection and abscess formation. Risk factors include poor oral hygiene, diabetes, immunocompromised states, and low socioeconomic status [4]. However, non-identified causes of pediatric deep neck infection varied between 35% and 40% [5]. Our patient denied any history of pharyngeal trauma prior to this presentation, although the dental examination showed no evident pathology.

The most common clinical presentation was neck stiffness and odynophagia, and the most common physical findings were fever, stiff neck, bulging of the oropharyngeal wall, a neck mass, and lymphadenopathy [3]. The palate is usually not edematous, and unilateral tonsillar exudate is rare. Also, tonsillar medialization and trismus were uncommon findings in retropharyngeal abscesses [4]. A rapidly growing retropharyngeal abscess may lead to potential life-threatening dyspnea that requires rapid airway management [6]. In this present case, no bulging of the oropharyngeal wall was noted, as the abscess was below the level of the tongue base, no edema was observed in the palate, and no trismus. At the same time, tonsils showed no hypertrophy, exudate, or medialization.

For radiological assessment, lateral neck radiographs are used for initial evaluation, whereas CT with intravenous contrast is the gold-standard imaging modality for suspected retropharyngeal abscesses [4]. In our patient, the images were consistent with an abscess, as they showed increased thickness of the prevertebral space, fluid collections, and gas bubbles within it, which partially obstructed the airway.

With respect to the treatment, empirical intravenous broad-spectrum antibiotic therapy is recommended to cover both aerobic and anaerobic pathogens until culture results are available [1]. As reported in the study [7], which included 30 patients diagnosed with retropharyngeal and parapharyngeal abscesses, 16 had abscesses with diameters greater than 25 mm. Still, all of them were treated with intravenous antibiotic and corticosteroid therapy for 48-72 hours, resulting in marked symptom improvement and a reduced length of stay. In agreement with the previous study, our patient received intravenous antibiotics and corticosteroid therapy and showed significant improvement; however, unlike their cases, surgical drainage of the abscess was also performed, which may have contributed to optimal recovery.

Surgical treatment with abscess drainage can be performed, especially in patients with compromised airways or when the abscess exceeds 3 cm in size [8]. It is usually accomplished through an intraoral approach, which avoids scarring on the neck. In complicated cases, an external cervical approach can be performed, as the intraoral approach can be dangerous or impossible to use when the abscess is deep or located in the posterior region [1,7]. In our case, the retropharyngeal abscess extended into the mediastinum, so we chose an external cervical approach to ensure complete drainage.

The absence of culture growth limits the present case despite a positive swab result, which might have been influenced by prior antibiotic therapy or technical factors. Regardless, in this study, we emphasize the importance of early diagnosis and treatment of retropharyngeal abscess, as it may lead to serious complications, including significant airway obstruction, mediastinitis, erosion of the carotid artery, and jugular vein thrombosis [1,3].

## Conclusions

This case underscores the importance of rapid assessment and structured management of retropharyngeal abscess in an adolescent patient who presented with dyspnea, rapidly progressive dysphagia, and high-grade fever. Imaging showed a retropharyngeal abscess with an intrathoracic extension. In this reported case, the absence of a complete microbiological analysis represents a limitation. Despite this limitation, our patient was effectively treated with intravenous antibiotics and corticosteroid therapy, along with external cervical incision and drainage.
